# Management of airway complications following lung transplantation: first interventional bronchoscopy report from Türkiye

**DOI:** 10.55730/1300-0144.5830

**Published:** 2024-05-23

**Authors:** Efsun Gonca UĞUR CHOUSEIN, Demet TURAN, Mustafa VAYVADA, Elif TANRIVERDİ, Ahmet Erdal TAŞÇI, Mehmet Akif ÖZGÜL, Erdoğan ÇETİNKAYA

**Affiliations:** 1 Division of Interventional Pulmonology, Department of Pulmonology, Yedikule Chest Diseases and Thoracic SurgeryEducation and Research Hospital, University of Health Sciences, İstanbul, Turkiye; 2 Division of Lung Transplantation, Department of Thoracic Surgery, Kartal Kosuyolu Training and Research Hospital, University of Health Sciences, İstanbul, Turkiye; 3 Division of Interventional Pulmonology, Department of Pulmonology, Başakşehir Çam and Sakura City Hospital, University of Health Sciences, İstanbul, Turkiye

**Keywords:** Lung transplantation, airway complications, interventional bronchoscopy

## Abstract

**Background/aim:**

Lung transplantation is the treatment of last resort for many chronic lung diseases. Airway complications (AC) following lung transplantation, such as bronchial stenosis, dehiscence, malacia, and fistula, account for frequent hospital admissions, additional treatment costs, decreased quality of life, and reduced survival rates. Beyond surgical and medical preventive efforts, interventional bronchoscopy (IB) can be used in the management of these complications. The aim of the study is to evaluate the efficacy of IB on the management of AC following lung transplantation.

**Materials and methods:**

A retrospective analysis was done using the data of lung transplant patients with AC referred to the interventional pulmonology unit between December 2012 and December 2019.

**Results:**

From a total of 116 lung transplants, the data of 14/116 (12%) patients and 14/220 (6.3%) anastomoses in the same lung transplant group with AC requiring IB were analyzed. In these 14 patients, the diseases leading to lung transplantation were interstitial lung diseases (ILD) (50.0%), bronchiectasis (28.6%), pulmonary arterial hypertension (PAH) (7.1%), chronic obstructive pulmonary disease (COPD) (7.1%), and COPD + bronchiectasis (7.1%). Airway stenosis was the most common airway complication, and it developed mostly in the right bronchial system.

The 14 patients underwent 27 total sessions of IB with an average of 2–3 per patient. Airway patency was successfully achieved in 74.1% of the procedures. Mechanical dilatation with a balloon and/or a rigid tube was the most preferred procedure (81.5%). Permanent airway patency was achieved in eight (57.4%) patients. No early complications were encountered (0%). The late complication rate was 48.1%. The most frequent late complication was restenosis, which cannot be directly attributed to IB.

**Conclusion:**

IB is safe to perform on lung transplant patients with AC. It has low procedural complication rates and can be performed repeatedly. Because of the high rate of restenosis, interventional pulmonologists should find out treatment modalities with lower rates of restenosis.

## 1. Introduction

Lung transplantation is the final treatment option for many chronic lung diseases such as interstitial lung diseases (ILD), chronic obstructive pulmonary disease (COPD), bronchiectasis, and pulmonary arterial hypertension (PAH) [1,2].

Complications following lung transplantation can be categorized into early or late, pulmonary, or extrapulmonary, and they can lead to frequent hospital admissions, additional treatment costs, decreased quality of life, and reduced survival rates. Airway complications (AC), including bronchial stenosis, bronchial dehiscence, obstructive granulation tissue formation, bronchomalacia, and bronchial fistula, have a 15%–20% rate of occurrence following lung transplantations, causing further respiratory symptoms and pulmonary function abnormalities [1,3–7].

These complications stem from the surgical technique of lung transplantation, which differs from the other solid organ transplantations. Early ischemia, inflammation, necrosis, or infection may develop both at the site of the anastomosis and in the distal parts, because systemic arterial blood supply cannot always be routinely provided during the procedure [4,8].

AC can develop even with the use of new surgical techniques, repair surgeries, and medical treatments. In these situations, interventional bronchoscopy (IB) can be used for treatment [1,9,10].

The preferred IB procedures vary according to the clinic, the location of the AC, its severity, the experience of the interventional pulmonologist, and the available resources of the center. Mechanical methods, such as mechanical dilatation with a rigid tube and/or balloon, mechanical resection, and airway stenting, and thermal methods (preferably cryotherapy), are the most preferred IB procedures. These methods can be used as a single or multimodal treatment [1].

The aim of this study is to evaluate the efficacy of IB in the management of AC following lung transplantation by examining the bronchoscopic findings, preferred methods, early and late complications of procedures and follow-up results.

## 2. Materials and methods

The data of lung transplant patients with AC who were referred to the interventional pulmonology unit between December 2012 and December 2019 were retrospectively analyzed. The patients’ bronchoscopic findings, preferred treatment methods, and treatment outcomes were reviewed. In accordance with the Declaration of Helsinki and hospital procedures, written and verbal consent was received from the patients, and the study was approved by the Ethics Committee of University of Health Sciences Turkey, Hamidiye Faculty of Medicine, with registration 20/130 and decision number 46418926- 050.03.04.

### 2.1. Interventional bronchoscopy procedures

IB procedures were performed under general anesthesia with rigid bronchoscopes. All patients were monitored using electrocardiography, their SpO2 values were tracked, and their arterial blood pressure was measured every 5 min. General anesthesia induction was achieved with midazolam 0.05–0.10 mg/kg, diprivan (maximum dose 1000 mg), remifentanil (maximum dose 2 mg), and rocuronium (maximum dose 50 mg), with the dosage adjusted to the patient’s condition. The equipment used for the IB procedures were the Dumon Series II rigid bronchoscopes (Efer Endoscopy, La Ciotat, France) with an optical system. Argon plasma coagulation (APC, 40 W, blended mode/continuous flow) was applied using an instrument from ERBE Elektromedizin GmbH (Tubigen, Germany). Endoluminal treatment was accomplished with a diode laser operating at a wavelength of 980 nm and 4–25 W in pulsed mode (Biolitec Cerals D25; Germany). Cryotherapy was performed using the ERBOKRYO system (ERBE Elektromedizin GmbH, Tubingen, Germany).

Treatment methods such as mechanical dilatation with/without a balloon, mechanical resection, APC, cryotherapy, and airway stenting were used. Control surveillance bronchoscopies and patient follow-up were performed in the transplantation center at intervals of 1, 3, 6, 9, and 12 months after treatment. Those who needed repeated IB procedures during the follow-up were referred back to our center.

Early complications related to the IB procedures were defined as those seen during the procedure or within 24 h after the procedure; these included the inability to wean from mechanical ventilation, bleeding, perforation of the bronchus, or arrhythmias. Late complications were defined as those seen 24 h or more after the procedure and included stent-related complications, granulation tissue formation, restenosis, and fistulization.

Procedural success was defined as the achievement of airway patency above 50%. Permanent airway patency was defined as airway patency that stayed above 50% during the full 12 months of the follow-up period.

### 2.2. Statistical analysis

The R software was used for statistical analysis of the data. Continuous variables were evaluated as mean ± standard deviation or as median (minimum–maximum) if they fit the normal distribution, and as a percentage (%) if they did not.

## 3. Results

### 3.1. Characteristics of the study population

**Figure 1:**
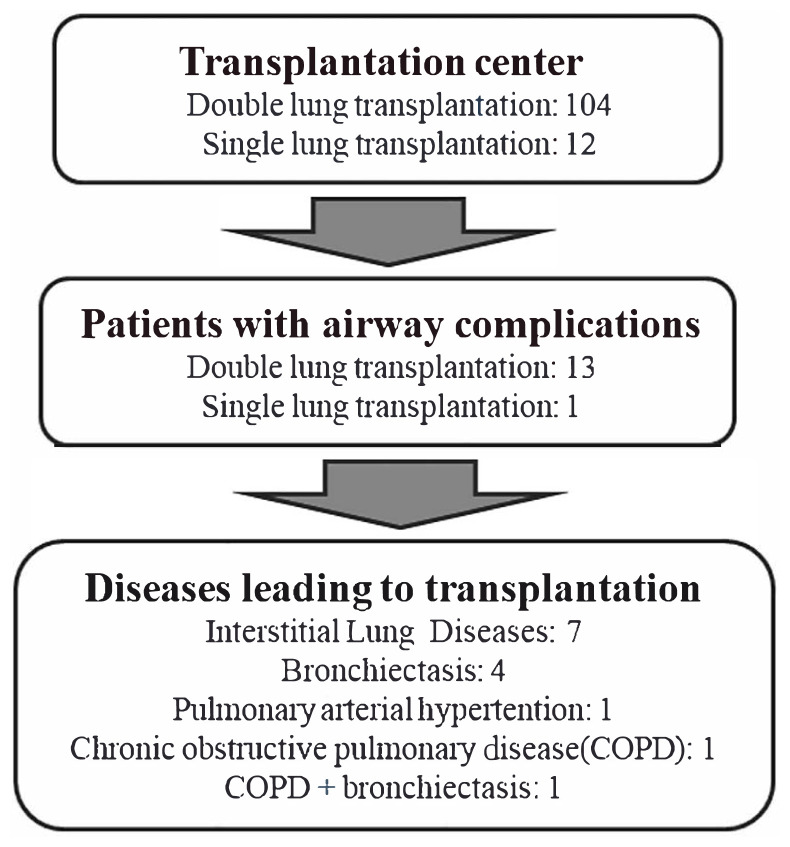
Flowchart of the study population.

During the study period, 104 double lung transplantations and 12 single lung transplantations, resulting in a total of 220 anastomoses, were performed on 116 patients in the transplantation center. AC were detected in 14 (12%) of the transplanted patients, of whom 13 (92.9%) had a double lung transplantation and one (7.1%) had a single lung transplantation. The AC rate requiring interventional bronchoscopy was 14/220 (6.3%) based on the total number of anastomoses. Eleven (78.5%) of the patients were male. The mean age of the study population was 41 ± 16.13 years (Table 1). In the 14 patients with AC, the diseases leading to lung transplantation were interstitial lung diseases (ILD) in seven (50.0%), bronchiectasis in four (28.6%), pulmonary arterial hypertension (PAH) in one (7.1%), chronic obstructive pulmonary disease (COPD) in one (7.1%) and COPD + bronchiectasis in one (7.1%) (Table 1, Figure 1).

**Figure 2:**
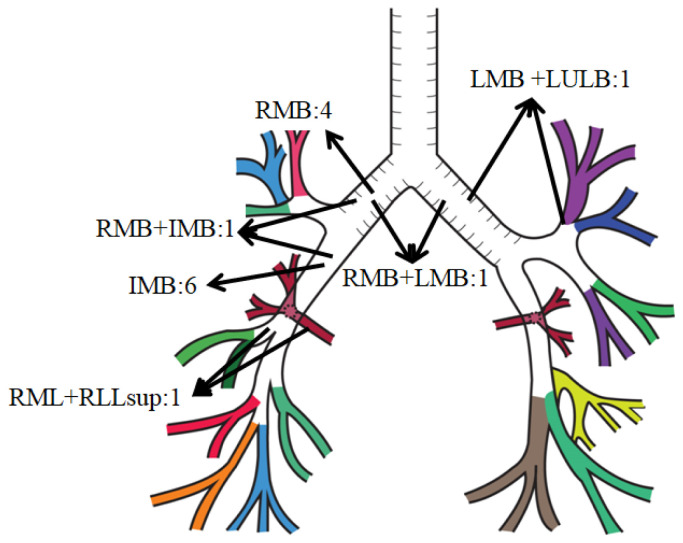
Distribution of the locations of the airway stenosis on the representative bronchial tree.

All 14 AC patients had airway stenosis, affecting the right bronchial system in 12 patients, the left in one patient, and both sides in another. Twelve patients had unilateral stenosis of the right bronchial system. The location of the stenosis was at the right main bronchus (RMB) at the site of the anastomosis in four, the intermediary bronchus (IMB) in six, the combined IMB and RMB (IMB + RMB) in one, and the combined right middle lobe bronchus (RML) and lower lobe superior segment bronchus inlet (RLL sup) (RML + RLL sup) in one. The site of the stenosis in the patient with left unilateral stenosis included both the left main bronchus (LMB) and the left upper lobe bronchus (LUL) (LMB + LUL). One patient had bilateral stenosis, detected at the anastomosis sites of the right main bronchus (RMB) and left main bronchus (LMB). The mean obstruction rate of stenosis detected in the bronchial lumens was 82.14 ± 12.36%. In addition to stenosis, nine (64.3%) patients had granulation tissue and two (14.3%) had malacia (Table 1, Figure 2).

The median time for AC detection by the transplantation center was 3.35 (3.4) (1.1–8.2) months, and the median time of admission to our center was 4.3 (2.3–22.4) months.

### 3.2 Interventional bronchoscopy procedures

**Figure 3:**
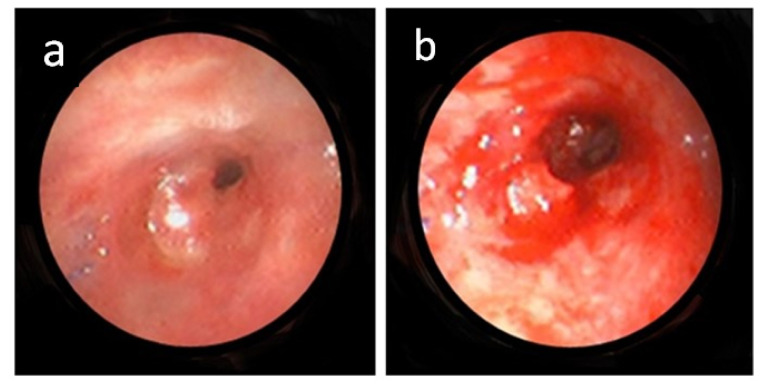
Bronchoscopic images of a patient with stenosis at the level of anastomosis level before (a) and after (b) a successful mechanical dilation.

A total of 27 IB procedures were performed: 14 for initial stenosis, nine for restenosis, two for granulation tissue, and two for stent removal. Nine (64.3%) of the 14 patients had multiple procedures. Mechanical dilatation with a balloon and/or rigid tube was the most common, comprising 22 (81.5%) of 27 procedures (Figures 3a and 3b). The other procedures included cryotherapy (8, 29.6%), mechanical resection (5, 18.5%), APC (3, 11.1%), and airway stenting (3, 11.1%). These were applied as single or multimodal treatments (Table 2).

Among five (35.7%) patients who required airway stenting, two had anatomical incompatibility precluding stent placement. Oki stents (small Y-shaped stents specially designed for the right bronchial system) were successfully placed in the remaining three. One of the patients who was mechanically ventilated was successfully extubated following the stent placement and was followed without any stent-related complications during 12-month follow-up period. One patient presented with stent migration and one with granulation tissue formation obliterating the stent lumen after a median of 22.5 (15–30) days. These patients had their stents removed on the 48th and 82nd days, respectively.

The procedural success rate of all IB procedures was 20/27 (74.1%). Adequate dilatation and/or stenting could not be achieved in 7/27. Reasons for insufficient dilatation or stenting failure were stenosis presenting out of reach of the rigid bronchoscope, uncertainty about the patency of the airways behind the stenosis, and anatomical incompatibility of airways for stenting. Thirteen (48.1%) of the 27 procedures were repeated procedures. Nine (69.2%) of the repeated procedures were performed for restenosis, two (15.4%) for granulation tissue formation, and two (15.4%) for stent removal (Table 2).

No procedural or early complications related to the IB were encountered (0%). The rate of late complications was 13/27 (48.1%). The late complications included restenosis, which could not be directly attributed to the IB, granulation tissue formation, and stent-related complications. Two airway stents were removed earlier than planned due to migration and granulation tissue obliterating the stent lumen; these two patients did not experience restenosis in the remaining follow-up period.

Permanent patency was achieved in 8/14 (57%) patients. Five patients required multiple procedures to achieve permanent patency. In two of the remaining six patients, airway patency was achieved temporarily during the first procedure but could not be maintained.

Scanning of the death notification system revealed that six (42.9%) patients were still alive at the end of the period covered by the study. For the other eight, the causes of death were rejection in 4/8, pulmonary infection in 2/8, and extrapulmonary comorbidities in 2/8.

## 4. Discussion

AC is an important cause of morbidity and mortality for lung transplant patients at varying rates depending on the number of cases or anastomoses. Olland et al. and Faccioli et al. state that new surgical anastomosis techniques, novel immunosuppressive agents, and repair surgeries demonstrate improved effectiveness in both prevention and treatment of AC. However, AC can still occur and IB procedures are needed for management [9–12].

The present study, which provides the first data from Türkiye on this subject, shows that IB is safe in the management of AC following lung transplantations. It has low early complication rates and can be performed multiple times. IB can provide and maintain airway patency in these susceptible group of patients.

According to Frye et al., there is no association between the primary disease leading to lung transplantation and the development of AC [6]. Although the number of patients with COPD and ILD among transplant recipients and on the transplant center’s waiting list were similar [13], AC following lung transplantation was significantly more common in ILD patients than in COPD patients. The related pathophysiology has not been definitely established as multivariate analyses are needed to statistically elucidate the relation between the underlying disease and the development of airway complications, as well as the predicted risk factors.

Studies by Mazzetta, Frye, and Thistlethwaite, among others, state that the most common AC following lung transplantation is airway stenosis, followed by granulation tissue formation and malacia [4,6,12,14,15]. We also observed that airway stenosis and granulation tissue formation were the most frequent AC.

In both single and double lung transplanted patients, AC develops most frequently in the right bronchial system. Patoir et al. reported that complications such as ischemia, necrosis, and stenosis were more common at the anastomotic level in the right lung. AC tends to affect the right bronchial system because the vascular structures that supply this region are located deeper in the parenchyma than their left counterparts, leading to difficulties in surgical anastomosis [4,6,8,9,15–17]. This study found that airway stenosis was most frequently observed in the right bronchial system and at the anastomosis level.

AC following lung transplantations were managed by interventional pulmonology specialists primarily with mechanical dilatation. Patoir et al. preferred mechanical dilatation using rigid tubes, which they found safer for the anastomotic level. Nevertheless, dilatation alone was not sufficient in most cases, and multimodal treatment using airway stents were applied. Stents were also the treatment of choice in the studies of Samano and Dibardino [1,12,15,18–20]. In this study, mechanical dilatation using only a rigid tube or accompanied by balloon dilatation was the most frequently preferred treatment.

Patients frequently develop restenosis following dilatation procedures and require recurrent interventions. Samano and DiBardino reported that they were able to provide rapid airway patency using balloon dilatation, requiring 2–5 repetitive procedures per patient for restenosis. They reported a success rate of 80% in establishing airway patency, and a restenosis rate of 52% [18–20]. In this study, airway patency was achieved in 74.1% of 27 procedures. An average of 2–3 mechanical dilatations were performed per patient, which comprised 85.1% of the total procedures.

To the best of our knowledge there are no clear data in the literature regarding the success rates of IB in maintaining permanent airway patency. In this study, permanent airway patency was achieved in 57% of patients for the full follow-up duration of 12 months.

Patients with early postoperative complications that could be managed surgically or by flexible bronchoscopy alone, such as bronchial anastomotic leakage and dehiscence, were primarily managed at the transplant center. The patients requiring IB procedures despite these interventions were referred to our center. The reported rate of early complications following IB is low. Late complications such as restenosis, granulation tissue formation, and those related to the airway stent are more common [4,6]. No procedure-related complications were observed in this study, and the same early and late complications were found as stated in the literature. Complications could potentially be reduced with the use of 3D personalized stents, biodegradable stents, or drug-eluting stents containing agents such as sirolimus, everolimus, zotarolimus, and pirfenidone. More extensive use of these stents for other pulmonary conditions might open the way for their future use in lung transplant patients [21–26]. It seems that the short-term use of airway stents could be possible for lung transplant patients since two patients in this study did not develop any restenosis following early stent removal, as mentioned in a recent review [27].

A major limitation of this study is its retrospective structure with a small number of patients. In addition, because all patients had to be referred back to the transplantation center after their IB treatment, the results of pulmonary function tests and quality of life questionnaires, which could objectively show the success of the procedures, could not be obtained. On the other hand, lung transplant patients comprise a susceptible patient group that requires specialized care in follow-up and complication management. Being the first data from Türkiye about the management of AC following lung transplantations, this study can guide interventional pulmonologists, pulmonologists, and thoracic surgeons who work in this field.

In conclusion, IB procedures performed on lung transplantation patients with AC are determined to be safe. They have low early complication rates and can be performed multiple times. Interventional pulmonologists should develop new strategies and find new treatment options for the prevention of restenosis in lung transplant patients.

## Figures and Tables

**Table 1:** General characteristics of lung transplant patients with airway complications.

Variables	n	%
**Total number of patients**	14	100
**Sex (male/female)**	11/3	78.57/21.43
**Age (mean±SD) years**	41±16.13	
**Cause of lung transplantation**		
ILD	7	50.0
Bronchiectasis	4	28.6
COPD	1	7.1
PAH	1	7.1
COPD+Bronchiectasis	1	7.1
**Airway complications**		
Stenosis	14	100
Granulation	9	64.3
Malacia	2	14.3
**Localization of the AC**		
**Right bronchial system**	12	85.7
RMB	4	28.6
IMB	6	42.9
IMB+RMB	1	7.1
RML+RLL sup	1	7.1
**Left bronchial system**	1	7.1
LMB +LUL	1	7.1
**Bilateral**	1	7.1
RMB+LMB	1	7.1

Abbreviation: n: number, %: percentage, SD: standard deviation, ILD: interstitial lung diseases, COPD: chronic obstructive pulmonary disease, PAH: pulmonary arterial hypertension, RMB: right main bronchus, IMB: intermediary bronchus, RML: right middle lobe bronchus, RLL sup: right lower lobe superior, LMB: left main bronchus, LUL: left upper lobe.

**Table 2:** Interventional bronchoscopy procedures and outcomes.

Variables	n	%
**IB procedures**	27	100
Mechanical dilatation	22	81.5
APC	3	11.1
Cryo	8	29.6
Mechanical resection	5	18.5
Airway stenting	3	11.1
**Complications of IB**	13/27	48.1
**Early**	0	00.0
**Late**	13/27	48.1
Restenosis	9/13	69.2
Granulation tissue	2/13	15.4
Airway stent related	2/13	15.4

n: Number, %: percentage, IB: Interventional bronchoscopy, APC: Argon plasma coagulation.
